# The Role of DNA Methylation in Insulin Resistance and Metabolic Dysregulation in Polycystic Ovary Syndrome Patients: A Systematic Review

**DOI:** 10.7759/cureus.84279

**Published:** 2025-05-17

**Authors:** Lily Tehrani, Michelle Tashjian, Chetana Movva, Yaacov Fakiro, Joshua Frank, Lubov Nathanson

**Affiliations:** 1 Medicine, Nova Southeastern University Dr. Kiran C. Patel College of Osteopathic Medicine, Fort Lauderdale, USA; 2 Medicine, Nova Southeastern University Halmos College of Arts and Sciences, Davie, USA; 3 Medicine, Touro University Nevada College of Osteopathic Medicine, Henderson, USA; 4 Institute of Neuro-Immune Medicine, Nova Southeastern University Dr. Kiran C. Patel College of Osteopathic Medicine, Fort Lauderdale, USA

**Keywords:** androgen, dna methylation, epigenetics, granulosa cells, insulin resistance, mirna, pcos, transcriptome

## Abstract

Polycystic ovary syndrome (PCOS) is a multifactorial endocrine disorder characterized by hyperandrogenism, ovulatory dysfunction, and metabolic disturbances, with insulin resistance (IR) playing a critical role in its pathogenesis. Emerging evidence suggests that epigenetic mechanisms, such as DNA methylation, may influence insulin signaling and metabolic pathways in PCOS by altering gene expression without changing the DNA sequence. This systematic review aims to evaluate the impact of DNA methylation on IR and metabolic dysfunction among PCOS patients by analyzing key genes involved in these processes. A comprehensive literature search was conducted using the databases Ovid, EMBASE, and Web of Science to analyze studies published between 2010 and 2025. Studies that evaluated DNA methylation and its association with IR and metabolic parameters in PCOS patients were included. Eligibility criteria followed the PRISMA guidelines. A total of 10 studies met the inclusion criteria. Hypermethylation of insulin receptor (*INSR)* and lamin A/C (*LMNA)* was associated with reduced insulin sensitivity, while hypomethylation of insulin receptor substrate 1 (*IRS1)* and bone morphogenetic protein 4 (*BMP4)* led to increased gene expression, contributing to metabolic dysregulation through increased androgen production. Epigenetic alterations were observed in granulosa cells and skeletal muscle tissues, highlighting tissue-specific differences. However, variability in study design and small sample sizes limited the generalizability of these findings. DNA methylation is pivotal in IR and metabolic dysfunction in PCOS. Understanding these epigenetic modifications may provide insights into potential therapeutic targets and lifestyle modifications aimed at reversing gene expression abnormalities and improving metabolic outcomes in patients suffering from PCOS and other metabolic disorders.

## Introduction and background

Polycystic ovary syndrome (PCOS) is a complex endocrine disorder affecting women of reproductive age, with a broad impact on metabolic, reproductive, and endocrine systems [1]. Diagnosis is primarily established using the Rotterdam criteria, which state that PCOS is confirmed when two of the following are present: oligo-amenorrhea, hyperandrogenism (HA), and polycystic ovaries on ultrasonography [2]. Although the Rotterdam criteria can aid in the diagnosis of this condition, PCOS remains a complex disorder with a wide variety of presentations, often making it difficult to manage and identify efficiently [3]. Between 2006 and 2019, the overall incidence of PCOS was 42.5 per 10,000 person-years, and the prevalence in 2019 was 5.2% among women aged 16 to 40 years [4]. Nearly 50% of PCOS patients experience insulin resistance (IR) as a result of excess serine phosphorylation of the insulin receptor (*INSR*) [5]. IR refers to the body’s reduced sensitivity to insulin, where cells do not respond effectively to the hormone. This leads to elevated blood glucose levels and compensatory increases in insulin secretion to maintain glycemic control [6]. 

Severe IR results in overstimulation of the ovarian theca cells and suppression of the sex hormone-binding globulin (SHBG), leading to increased androgen production and higher levels of free (active) testosterone circulating in the blood. This upregulation of these hormones in women contributes to male-pattern symptoms, such as excessive hair growth and acne [7].

The effects of IR in PCOS patients also predispose them to type 2 diabetes mellitus (T2DM), hypertension, and related cardiovascular diseases. T2DM is a metabolic disorder characterized by impaired insulin sensitivity, leading to hyperglycemia [8]. Obesity, which is also associated with T2DM, exacerbates IR through the accumulation of excess adipose tissue, further worsening symptoms and accelerating disease progression [9]. Beyond metabolic and hormonal disturbances, underlying genetic factors, such as DNA methylation, have also been shown to play a significant role in the onset and progression of PCOS [10]. 

DNA methylation refers to the mechanism by which methyl groups are added to cytosines in CpG sequences. Methylation occurring in promoter regions of genes significantly reduces transcriptional activity, thereby silencing gene expression [11]. Methylation is a crucial epigenetic process in regulating gene expression patterns within the human genome and becomes heavily dysregulated in PCOS [12]. Studies have identified abnormal DNA methylation patterns in genes related to metabolic function, ovarian regulation, and insulin signaling pathways. Furthermore, dysregulation of genes involved in androgen synthesis, such as *CYP19A1*, also contributes to the hormonal imbalances observed in PCOS [13]. Understanding the role of DNA methylation in PCOS progression becomes essential for identifying biomarkers, which can support earlier diagnosis and guide gene-targeted therapies [14]. These therapies represent a promising future treatment, as epigenetic modifications are reversible and can be targeted to restore normal gene expression [15]. Further research in these areas may allow for opportunities to mitigate the long-term complications of PCOS, better understand its complexity, and potentially improve symptomology [16]. 

This review aims to evaluate the complex relationship between DNA methylation, IR, and metabolic dysregulation in PCOS patients. By examining the current evidence on epigenetic modifications in various tissues of PCOS patients, we seek to identify potential biomarkers and therapeutic targets. Moreover, this review will synthesize the latest findings on DNA methylation changes in PCOS, their association with IR and metabolic complications, and discuss the implications for future research and clinical practice. 

## Review

Methods 

Search Strategy 

A comprehensive systematic literature review was conducted using Ovid, EMBASE, and Web of Science. Articles were analyzed in a stepwise process. Two reviewers independently evaluated the title and abstract, and for included studies, proceeded to review the full-text manuscript. In the case of disagreement, the reviewers held a discussion to reach a consensus. If a consensus could not be reached, a third reviewer was consulted to resolve the discrepancy. The search strategy used Boolean operators ("AND" and "OR") between the selected keywords as follows: (“PCOS” OR “Polycystic Ovary Syndrome”) AND (“DNA methylation” OR “epigenetics”) AND (“insulin resistance” OR “metabolic dysfunction”). To ensure relevancy, only articles published between 2010 and 2025 were included. The Nova Southeastern University (NSU) library database was used for articles that were not freely accessible. 

Selection Criteria 

**Figure 1:**
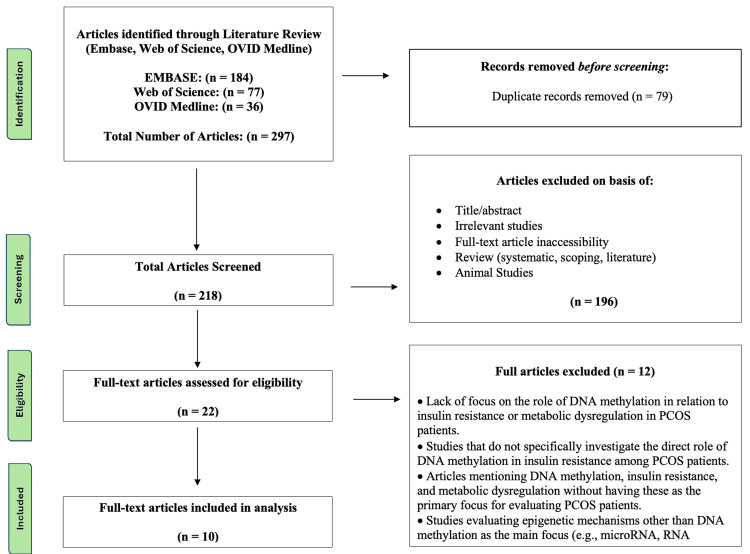
PRISMA diagram PRISMA: preferred reporting items for systematic reviews and meta-analyses.

Studies were deemed eligible for inclusion if they explored the relationship between epigenetic modifications, such as DNA methylation, and PCOS. Acceptable study types included randomized controlled trials, case-control studies, cross-sectional studies, and retrospective cohort studies. To be included, studies needed to be written in English, contain relevant keywords in the title or abstract, and assess the link between methylation and IR or metabolic markers. Studies were excluded if they involved non-PCOS populations, researched other forms of epigenetic changes (unless directly linked to DNA methylation), were literature reviews, systematic reviews, scoping reviews, commentaries, editorials, or animal studies. Duplicate studies were removed using Rayyan. The Preferred Reporting Items for Systematic Reviews and Meta-Analyses (PRISMA) was used to develop a flow diagram of the selection criteria for reproducibility [17] (Figure 1). 

The quality of the articles was determined using quality assessment and analysis tools, including the Joanna Briggs Institute (JBI) critical appraisal tools for case-control, cross-sectional, and quasi-experimental studies [18], as well as the Appraisal tool for Cross-Sectional Studies (AXIS) [19] for assessing cross-sectional study designs. Two reviewers independently reviewed each article and categorized the risk of bias as either low, moderate, or high based on the proportion of criteria met on the corresponding appraisal checklist. A study was considered low risk if ≥80% of items were marked “Yes,” moderate risk if between 50% and 79%, and high risk if <50%. Only studies where both reviewers assigned a score of 75% or higher “Yes” responses on the risk of bias checklist were included in the final synthesis. A summary of the risk-of-bias assessment for the 10 included studies is presented in Table 1. 

**Table 1:** Summary of risk-of-bias assessment for included studies Risk of bias was assessed using the JBI critical appraisal checklists for case-control, cross-sectional, and quasi-experimental studies, and the Appraisal Tool for Cross-Sectional Studies (AXIS). Ratings were based on the proportion of “Yes” responses to checklist items. Only studies scoring ≥75% were included in the final synthesis. JBI: Joanna Briggs Institute.

Study (Author, Year)	Study Design	Tool Used	% “Yes”	Risk of Bias
Zhong et al. (2021) [20]	Case-control	JBI (Case-Control)	80%	Low
Ting et al. (2013) [21]	Case-control	JBI (Case-Control)	80%	Low
Cao et al. (2021) [22]	Cross-sectional observational study	JBI (Cross-Sectional Studies)	75%	Moderate
Bhingardeve et al. (2025) [23]	Case-control	JBI (Case-Control)	90%	Low
Nilsson et al. (2018) [24]	Case-control	JBI (Case-Control)	100%	Low
Zhao et al. (2017) [25]	Cross-sectional	AXIS	95%	Low
Jones et al. (2015) [26]	Case-control	JBI (Case-Control)	100%	Low
Gao et al. (2024) [27]	Cross-sectional	JBI (Cross-sectional)	87.5%	Low
Shen et al. (2013) [28]	Cross-sectional	JBI (Cross-sectional)	75%	Moderate
Furtado et al. (2024) [29]	Quasi-experimental Study	JBI (Quasi-experimental)	80%	Low

Results

Zhong et al. (2021) conducted a case-control study to investigate the role of DNA methylation of *INSR* and anti-Mullerian hormone receptor type II (*AMHRII*) in the pathogenesis of PCOS [20]. Seventy-five PCOS patients between the ages of 20 and 39 years diagnosed by the Rotterdam criteria were compared to 20 healthy controls. The homeostatic model assessment of insulin resistance (HOMA-IR) was calculated, with IR defined as a HOMA-IR >2.6. Western blot analysis revealed a significant increase in *AMHRII* protein in the granulosa cells (GCs) of the ovaries of PCOS patients (P = 0.002). Conversely, *INSR* expression was significantly lower in the endometrium of PCOS patients compared to controls (P = 0.036). DNA methylation revealed that the Pos.3 CpG site of the *AMHRII* gene was hypomethylated in PCOS patients (P < 0.05), whereas the Pos.4 site was hypermethylated (P < 0.05). Although the precise genomic locations of these CpG sites weren’t specified, the methylation patterns may influence transcriptional activity. Histopathological analysis of PCOS ovaries demonstrated a thickened tunica albuginea, stromal hyperplasia, and abnormal follicle development. Additionally, the endometrium of these patients exhibited epithelial hyperplasia, glandular distortion, and stromal hyperplasia. Clinically, PCOS patients had lower follicle-stimulating hormone (FSH) levels and higher luteinizing hormone (LH), LH/FSH ratio, testosterone, anti-Mullerian hormone (AMH), and HOMA-IR values compared to controls (P < 0.05). The study reported that methylation levels of the *AMHRII* gene (r = 0.532, P = 0.000) and *INSR* gene (r = 0.281, P = 0.03) were significantly associated with IR and clinical characteristics in PCOS [20].

Ting et al. (2013) studied the correlation between IR and CpG island methylation of the lamin A/C (*LMNA*) gene in PCOS patients through a molecular case-control study [21]. Genome-wide methylation microarray screening was performed on peripheral blood mononuclear cells from three IR-PCOS patients and one healthy control to identify candidate genes associated with IR in PCOS, including *LMNA, RPS4X,* and *KCNJ11*. *LMNA* was selected for further validation, as *RPS4X* and *KCNJ11 *did not yield significant findings. To validate these findings, the promoter region methylation of *LMNA* was examined in a larger cohort of 24 IR-PCOS women and 24 age-matched normal controls. The findings identified general hypermethylation of the CpG island in the promoter region of the *LMNA* gene among PCOS women. Of the 20 CpG sites analyzed, 12 had significantly altered methylation levels (P < 0.05) between PCOS patients and controls. Specifically, the third and fourth CpG sites within the first promoter-associated fragment averaged 0.7933 ± 0.0046 and 0.9075 ± 0.0012 in PCOS females, compared with 0.7448 ± 0.0059 and 0.8304 ± 0.0032 in controls (P < 0.005). In addition, locations four, five, six, seven, nine, 10, 11, 12, 14, and 15 within a second promoter-associated fragment also exhibited significantly different methylation levels between groups (P < 0.005). These findings suggest that hypermethylation of the *LMNA* promoter is associated with IR in PCOS patients, indicating that *LMNA* may play a role in the regulation of PCOS-associated IR [21].

Cao et al. (2021) investigated the role of DNA methylation in PCOS pathogenesis by comparing GC methylation profiles between PCOS patients and healthy controls in an observational study [22]. The results demonstrated highly reduced levels of global DNA methylation in PCOS GCs, particularly within the first intronic regions of genes. Transcriptome analysis revealed 470 upregulated and 548 downregulated genes in PCOS GCs, and pathway enrichment analysis identified significant dysregulation of IR, adipocyte differentiation, and steroid metabolism. Key genes within these pathways included *PCK1*, which is involved in gluconeogenesis, and *CYP1B1*, which is involved in estrogen metabolism. Altered methylation of these genes may link their dysregulated expression to obesity and hormonal imbalances in PCOS. Further integrated analysis of DNA methylation, mRNA, and microRNA (miRNA) expression data revealed a coregulated network involving bone morphogenetic protein 4 (*BMP4*)*, *insulin receptor substrate 1 (*IRS1*)​​​​​​, and *ETS1*. Researchers identified 495 hypomethylated and 25 hypermethylated differentially methylated regions (DMRs), with the majority of hypomethylated DMRs located within the promoter region and first intron. The study also found 19 upregulated and 10 downregulated miRNAs in PCOS GCs. In particular, miR-141-3p and miR-508-3p were involved in regulatory pathways including transforming growth factor-beta 1 (TGF-β1), mitogen-activated protein kinase (*MAPK*)*, *tumor necrosis factor (*TNF*), and circadian rhythms. Additionally, 13 genes were co-regulated by both miRNAs and hypomethylation, including *BMP4, IRS1*​​​*, ETS1*​​​​, *FGFR1, CYP1B1,* and *KLF5*. Specifically, genes involved in lipid metabolism, steroid synthesis, and glucose metabolism were regulated by abnormal DNA methylation, which may be implicated in the pathogenesis of PCOS [22].

Bhingardeve et al. (2025) explored the role of DNA methylation in regulating miRNA expression and its subsequent impact on target gene expression in the ovaries of PCOS women through a case-control study [23]. The study compared GC samples of PCOS patients with age- and body mass index (BMI)-matched controls to assess the abundance of transcripts from 14 miRNAs involved in ovarian processes while measuring their CpG-DNA methylation levels. Due to variability in nucleic acid yield, 20 samples from each cohort (PCOS and control) were included in the analysis. Participants underwent controlled ovarian hyperstimulation using the gonadotropin-releasing hormone antagonist protocol for in vitro fertilization, while recombinant hCG was administered to induce ovulation. Hypermethylation and downregulation of miR-10b-5p, miR-127-3p, miR-5189, miR-410-3p, and miR23a-3p were associated with upregulation of phosphatase and tensin homolog (​*PTEN*)​​​​​, matrix metallopeptidase 13 (*MMP13*), oxidized low-density lipoprotein receptor 1 (*OLR1), *tet methylcytosine dioxygenase 3 (*TET3*)*,* and apoptotic protease-activating factor 1 (*APAF1*) in PCOS. In contrast, hypomethylation and upregulation of miR-140-5p, miR-182-3p, miR-200b-5p, and miR-3687 were associated with downregulation of *FZD6, LRP6, ZEB1,* and low-density lipoprotein receptor (LDLR). A significant inverse correlation was also observed between DNA methylation levels and miRNA transcript levels (e.g., miR-10b-5p, miR-127-3p, miR-140-5p), as well as between miRNA levels and the expression of their respective target genes (e.g., *PTEN, MMP13, LDLR*). The study suggests that altered miRNA expression, along with accompanying DNA methylation changes, could be involved in reproductive and metabolic dysfunction in PCOS [23].

Nilsson et al. (2018) investigated genome-wide DNA methylation and gene expression profiles in skeletal muscle from 17 women with PCOS and 14 BMI-matched controls through a case-control study [24]. Eighty-five differentially expressed transcripts were identified in PCOS patients, including *DYRK1A, SYNPO2, SCP2*, and *NAMPT*, as well as reduced expression of immune-related gene sets, including HLA genes and pathways associated with antigen presentation and type 1 diabetes. While only two CpG sites (cg00973947 in c3orf58 and cg10074626 in an intergenic region) differed in DNA methylation, nearly 30% of differentially expressed genes revealed a correlation between mRNA expression and CpG site methylation within or near the gene. Most of these correlations were inverse, suggesting a suppressive regulatory role of DNA methylation on gene expression. Functional in vitro assays with human skeletal muscle cells indicated that insulin promoted glycogen deposition in myotubes, whereas testosterone inhibited collagen type I alpha 1 chain (*COL1A1*) and mitogen-activated protein kinase kinase 6 (*MAP2K6*) expression, suggesting insulin and androgen pathways in muscle metabolism. The researchers concluded that PCOS is associated with dysregulated skeletal muscle gene expression, disrupted signaling pathways, and modest alterations in DNA methylation patterns, which may contribute to the metabolic disturbances observed in affected women [24].

The cross-sectional study by Zhao et al. (2017) investigated the association between peroxisome proliferator-activated receptor gamma coactivator 1-alpha (PPARGC1A) promoter methylation, leukocyte mitochondrial DNA (mtDNA) content, and metabolic risk in 175 women with PCOS compared with 127 healthy controls [25]. The study found that women with PCOS had higher *PPARGC1A* promoter methylation (36.5% vs. 26.3%) and lower mtDNA content (mtDNA/nDNA ratio) compared to controls, even after adjusting for BMI (P < 0.0001). Women with PCOS also exhibited higher levels of insulin, IR, total testosterone, free androgen index, and triglycerides compared to the control group. Further stratification using adult treatment panel III criteria for metabolic syndrome revealed that PCOS patients with one or more risk factors (59.43%) had higher *PPARGC1A* methylation and lower mtDNA content than those without risk factors (41.57%). The study concluded that *PPARGC1A* methylation was significantly inversely correlated with mtDNA levels. These findings suggest that *PPARGC1A* promoter methylation and leukocyte mtDNA content could serve as potential biomarkers for assessing metabolic risk in women with PCOS [25].

Jones et al. (2015) conducted a cross-sectional observational systems genetics analysis, measuring gene expression and genomic DNA methylation patterns in adipose tissue biopsies from healthy controls and PCOS patients [26]. The researchers focused on 11 known PCOS risk loci identified through genome-wide association studies. Gene expression and DNA methylation patterns showed distinct, obesity-dependent molecular signatures. Specifically, in non-obese PCOS women, luteinizing hormone/choriogonadotropin receptor (*LHCGR*) was significantly overexpressed due to hypomethylation within the *LHCGR* locus. Increased expression of this gene suggests increased sensitivity to LH, potentially driving excess ovarian androgen secretion. In contrast, obese women with PCOS showed underexpression and hypermethylation at the *INSR* locus in adipose tissue, indicating impaired insulin signaling and IR. Downregulation of *WIBG, RAB5B*, and *IKZF4 *was also observed, although only LHCGR and WIBG remained statistically significant after false discovery rate (FDR) correction. Upon investigating methylation differences at specific loci, researchers found 17 CpG sites across these 11 known PCOS risk loci were identified as differentially methylated between patients and controls. Specifically, three CpG sites were hypomethylated within the *LHCGR* locus in non-obese patients, suggesting overexpressed *LHCGR* and increased sensitivity to LH. One CpG site was hypermethylated within the *INSR* locus in obese patients, corresponding with *INSR* underexpression and impaired insulin signaling. Finally, four CpG sites were differentially methylated within the *RAB5B* locus. Although not all CpG methylation changes remained statistically significant after multiple testing correction, several were associated with altered gene activity, suggesting their potential role in PCOS development [26]. 

Gao et al. (2024) conducted an observational case-control study to evaluate the epigenetic role of TGF-β1 methylation in women with PCOS and IR [27]. Heavy bisulfite sequencing was performed on DNA extracted from peripheral blood samples of PCOS patients with IR and HA and compared to controls with regular menstrual cycles and prior childbirth. The results indicated significant hypomethylation at CpG4 and CpG7 in the TGF-β1 gene promoter region in PCOS patients with IR compared to the control group. TGF-β1 methylation rate was negatively correlated to fasting insulin (FINS) (R = -0.32, P = 0.012) and HOMA-IR (R = -0.28, P = 0.029) and positively correlated with age (R = 0.38, P = 0.0032). This hypomethylation may influence TGF-β1 gene expression and contribute to the development of IR, a prominent feature of PCOS. Additionally, testosterone levels were found to be significantly higher in PCOS patients with high TGF-β1 methylation vs. those with low methylation (P < 0.001). However, no significant difference was found in mRNA or protein expression of TGF-β1 between the two groups, and no correlation was observed between methylation and protein expression. The findings demonstrate that TGF-β1 methylation status may serve as a potential biomarker for metabolic and hormonal risk in PCOS patients [27]. 

Shen et al. (2013) conducted a cross-sectional case-control study to analyze genome-wide DNA methylation differences in PCOS patients using methylated DNA immunoprecipitation (MeDIP) analysis [28]. The study included 15 participants: five PCOS patients with IR, five PCOS without IR, and five healthy controls. MeDIP analysis identified 40 differentially methylated genes between PCOS patients and controls, and 79 between non-IR and IR patients within the PCOS group. In particular, *CEBPB* was significantly differentially methylated between the PCOS-IR and non-IR groups (P = 0.00017). Researchers found that *CEBPB* was implicated in both regulatory and protein-protein interaction networks, suggesting its involvement in PCOS-related IR. Gene ontology (GO) and pathway enrichment analysis revealed differential methylation of immune response genes in the PCOS-IR group, including cytokine-cytokine receptor interaction, hematopoietic cell lineage, asthma, and the JAK-STAT signaling pathway. Cancer-related pathway genes were also enriched in both PCOS-IR and non-IR groups compared to controls. Clinically, PCOS patients exhibited significantly higher total testosterone, free testosterone, and antral follicle counts, along with lower SHBG levels compared to controls (P < 0.05). Additionally, IR patients demonstrated significantly higher FINS and HOMA-IR levels (P < 0.05) [28].

Furtado et al. (2024) conducted a retrospective analysis to study the influence of exercise on DNA methylation in 56 women with PCOS [29]. Participants were divided into two groups: 30 received supervised resistance training, and 26 patients received supervised aerobic training. Both groups trained three times per week for 50-60 minutes per session over 16 weeks. After 16 weeks, both exercise protocols resulted in improvements in anthropometric, metabolic, and hormonal parameters. Additionally, genome-wide DNA methylation levels increased following both types of training. However, the biological significance was limited, as the average change in beta values was small (<0.005). Resistance training affected all CpG contexts (islands, shores, shelves, and open sea), while aerobic training predominantly altered CpG sites in shores and open sea regions. Using an FDR threshold of 0.1, six DMRs were found in the resistance training group, while 14 were found in the aerobic group, all of which were hypermethylated. DMRs in the aerobic cohort were associated with *SNHG5*, *RPS27*, and *RIOK2*, whereas those in the resistance cohort were linked to *DSCR9*, *RPL14*, and *RHBDD3*. GO and pathway enrichment analysis revealed that aerobic DMRs are associated with RNA processing and ribonucleoprotein complex formation. Although aerobic and resistance training induced epigenetic changes, delta comparisons revealed no statistical difference in clinical outcomes between groups. The study used Horvath’s DNA methylation clock to predict biological age by evaluating methylation levels at 353 specific CpG sites and found no change in epigenetic age in either exercise group (P > 0.1). These findings suggest that increased genome-wide DNA methylation, particularly DMR hypermethylation, may be associated with the metabolic and hormonal improvements observed in PCOS patients following both types of exercise [29]. A summary of all included studies, including sample sizes, gene targets, metabolic markers, key findings, epigenetic impacts, and study limitations, is presented in Table 2.

**Table 2:** Characteristics of the studies examined AMHRII: anti-Mullerian hormone receptor type II; INSR: insulin receptor; HOMA-IR: homeostatic model assessment of insulin resistance; AMH: anti-Mullerian hormone; PCOS: polycystic ovary syndrome; IR: insulin resistance; NIR: non-insulin resistance; IRS1: insulin receptor substrate 1; LMNA: lamin A/C; FBG: fasting blood glucose; FINS: fasting insulin; GC: granulosa cell; MBD-seq: methyl-CpG binding domain sequencing; miRNA: microRNA; BMI: body mass index; PPARGC1A: peroxisome proliferator-activated receptor gamma coactivator 1-alpha; mtDNA: mitochondrial DNA; HA: hyperandrogenism; TGF-β1: transforming growth factor beta 1; NIR: non-insulin-resistant; LDL: low-density lipoprotein; Ox-LDL: oxidized low-density lipoprotein; LDLR: low-density lipoprotein receptor; HDL: high-density lipoprotein; HDL-C: high-density lipoprotein-cholesterol; DMRs: differentially methylated regions; SHBG: sex hormone-binding globulin; BMP4: bone morphogenetic protein 4; LPIN1: lipin-1; MMP13: matrix metallopeptidase 13; APAF1: apoptotic protease-activating factor 1; PTEN: phosphatase and tensin homolog; KLF10: Krüppel-like factor 10; COL1A1: collagen type I alpha 1 chain; MAP2K6: mitogen-activated protein kinase 6; OLR1: oxidized low-density lipoprotein receptor 1.

Title/Author	Sample Size	DNA Methylation Targets	Metabolic Marker	Key Findings	Epigenetic Impact	Study Limitations
Zhong et al. (2021) [20]	75 PCOS patients (aged 20 to 39 years) and 20 controls	Four methylation sites (Pos.1-Pos.4) were analyzed within the genes *AMHRII* and *INSR*	HOMA-IR: Levels were higher in PCOS patients (4.99 ± 2.76) vs. controls (1.65 ± 0.46); AMH: Levels were elevated in PCOS patients (8.10 ± 2.31 pg/mL) vs. controls (4.80 ± 2.15 pg/mL); Testosterone: Levels were higher in PCOS patients (3.09 ± 1.60 nmol/L) vs. controls (1.52 ± 0.88 nmol/L).	In PCOS patients, the gene expression of *AMHRII* was significantly higher than the control group (P = 0.002), while *INSR* gene expression was significantly lower in the endometrium (P = 0.036). No significant methylation differences were found at Pos.1 (P = 0.08) and Pos. 2 (P = 0.16) of the *AMHRII* gene. The PCOS group had a significantly lower level of methylation in Pos.3, but higher level in Pos.4 (P < 0.05). No significant methylation differences were found at Pos.1 (P = 0.08) and Pos.2 (P = 0.47) of the *INSR* gene. The PCOS groups had a significantly higher level of methylation in Pos.3 and lower in Pos.4 (P < 0.05). Significant correlations were found between *AMHRII* methylation and insulin receptor levels (P = 0.00), and between *INSR* methylation and insulin receptor levels (P = 0.03).	Abnormal DNA methylation at Pos.1 and Pos.4 of the *AMHRII* gene suggests enhanced protein expression and increased AMH levels; in PCOS patients, this methylation may alter gene activity by affecting transcription factor binding and DNA interactions; *INSR* gene methylation disrupts gene structure, leading to functional abnormalities and reduced receptor function; *INSR* dysfunction contributes to insulin resistance (IR) in PCOS; significant correlations were observed between gene methylation and IR.	Small sample size of 75 PCOS cases and 20 controls, which may lack the statistical power needed to generalize findings. All participants were recruited from a single hospital in Guangdong Province, restricting the study's ability to generalize findings to diverse populations such as those with different genetic backgrounds or environmental exposures. The study could not analyze DNA methylation in fresh ovarian and endometrial tissues due to the difficulty of collection.
Ting et al. (2013) [21]	Genome-wide methylation screening: 3 with IR and 1 control. Case-control study: 24 IR PCOS patients and 24 controls	20 CpG sites in the promoter region of the Lamin A/C (*LMNA*) gene	HOMA-IR: Elevated in PCOS patients (2.89 ± 0.57) vs. controls (1.58 ± 0.23) (P < 0.01). FBG: Higher in PCOS patients (7.21 ± 0.45 mmol/L) vs. controls (5.18 ± 0.26 mmol/L) (P < 0.01). FINS: Elevated in PCOS patients (16.34 ± 0.59 μIU/mL) vs. controls (5.78 ± 0.89 μIU/mL) (P < 0.01). Testosterone: Increased in PCOS patients (0.7 ± 0.08 nmol/L) vs. controls (0.3 ± 0.04 nmol/L) (P < 0.01).	All four of the CpG sites found in the *LMNA* gene fragment expressed significant increases in methylation in PCOS patients compared to controls (P < 0.05); 12 out of 20 CpG sites in the *LMNA* promoter region had significantly higher methylation levels in PCOS (P < 0.05). No significant methylation differences were found in *RPS4X* and *KCNJ11* genes, suggesting that *LMNA* hypermethylation is uniquely associated with PCOS-related IR.	The hypermethylation status of *LMNA’s* promoter region may contribute to reduced gene expression, affecting insulin sensitivity and metabolic regulation. Abnormal *LMNA* methylation can disrupt nuclear structure and chromatin interactions, leading to metabolic dysregulation.	Small sample sizes, particularly for the initial genome-wide screening (n=4), limit the study's statistical power and generalizability. The validation cohort (n=48) remains relatively small and lacks ethnic and geographic diversity. All participants were from a single clinic, which may introduce selection bias. The study lacks ethnic or geographic diversity, which could influence DNA methylation patterns.
Cao et al. (2021) [22]	GCs from PCOS patients and controls: RNA-Seq: 4 PCOS vs. 4 controls, MBD-seq: 3 PCOS vs. 3 controls, miRNA: 5 PCOS vs. 5 controls	*BMP4, IRS1, ETS1, KLF5, CYP1B1 *	Genes implicated in metabolic regulation, including *IRS1* and *LPIN1, *exhibited hypomethylation. Enriched metabolic pathways include insulin secretion, thyroid hormone signaling, and glucose metabolism, which contribute to IR and metabolic dysregulation.	DNA hypomethylation is a major characteristic in PCOS GCs, primarily in the first introns. Irregular DNA methylation and miRNA interactions disrupt normal ovarian function and metabolic regulation.	Hypomethylation in genes such as *IRS1* and *BMP4 *upregulates their expression, contributing to disrupted insulin signaling and fat metabolism; miRNAs, such as miR-141-3p, regulate target genes involved in PCOS pathogenesis, worsening metabolic and hormonal imbalances.	The sample size for each dataset was relatively small, which may limit the generalizability of the results. The study focused solely on GCs, which do not capture the full complexity of PCOS pathogenesis across different tissues.
Bhingardeve et al. (2025) [23]	25 women with PCOS and 25 age- and BMI-matched controls were recruited, but only 20 per group were used for methylation analysis due to sample quality issues	Hypermethylated and downregulated: miR-10b-5p, miR-23a-3p, miR-127-3p. Hypomethylated and upregulated: miR-140-5p, miR-182-3p, miR-200b-5p	Hypermethylation of miR-10b-5p was linked to ​*PTEN* overexpression, inhibiting insulin signaling and contributing to IR. Hypermethylation of miR-5189 was linked to increased OLR1, which binds oxidized LDL and promotes its breakdown through cellular uptake of the oxidized low-density lipoprotein (ox-LDL)-OLR1 receptor ligand complex. Hypomethylation of miR-3687 was linked to reduced LDLR, contributing to dyslipidemia.	Altered miRNA methylation profiles in GCs correlate with disrupted ovarian function and metabolic dysregulation. Changes in DNA methylation patterns regulate miRNA expression, which in turn affects target gene expression critical to PCOS pathophysiology.	Hypermethylation of miRNA promoter regions suppresses expression, leading to the overexpression of targets, including matrix metallopeptidase 13 (*MMP13)*, which is linked to ECM remodeling and follicular defects, and apoptotic protease-activating factor 1 (*APAF1)*, which promotes GC apoptosis.	The single time point design of the study prevents the establishment of direct causal relationships between DNA methylation of genes, such as *PTEN*, and gene expression changes in PCOS. The study relied on a relatively small cohort of 20 controls and 20 women with PCOS, limiting the generalizability of the results. Due to the pilot nature of the study, further evaluation through larger, diverse populations needs to be investigated.
Nilsson et al. (2018) [24]	17 women with PCOS and 14 age-, weight-, and BMI- matched controls	*KLF10*, *COL1A1*, and *MAP2K6* showed DNA methylation changes correlated with altered gene expression. Two CpG sites (cg00973947, cg10074626) were differentially methylated (q < 0.05)	HOMA-IR was higher in PCOS patients (3.06 ± 2.10) than in controls (2.02 ± 1.02), but was not significant (P = 0.138). C-peptide was significantly higher in PCOS patients (1.18 ± 0.46 ng/mL) than in controls (0.86 ± 0.35 ng/mL, P = 0.033), and the C-peptide index was also higher in PCOS (8.16 ± 3.41) vs. controls (5.74 ± 2.63, P = 0.035); FBG was lower in PCOS patients (4.87 ± 0.34 mmol/L) compared to controls (5.13 ± 0.44 mmol/L, P = 0.088, not significant). Triglycerides were higher in PCOS (1.17 ± 0.52 mmol/L) vs. controls (0.86 ± 0.31 mmol/L, P = 0.050, borderline significant). Testosterone was significantly higher in PCOS (446.2 ± 185.8 pg/mL) than in controls (262.0 ± 70.9 pg/mL, P = 0.001). Adipocyte size was also increased in PCOS and correlated with gene expression changes.	Women with PCOS had atypical skeletal muscle gene expression and DNA methylation changes; IR was found to be linked to transcriptional dysregulation; *KLF10* expression was upregulated in PCOS muscle cells and influenced by insulin. Testosterone downregulated *COL1A1* and *MAP2K6* expression in muscle cells. Epigenetic modifications contributed to altered immune system pathways in PCOS.	30% of differentially expressed genes correlated with DNA methylation changes, affecting insulin signaling and immune pathways; *KLF10* was linked to disrupted glucose metabolism; *COL1A1* and *MAP2K6* were downregulated by testosterone, impacting inflammation and ECM remodeling.	The relatively small sample size of 17 women with PCOS and 14 controls may limit the statistical power and generalizability of the findings. The study focused on skeletal muscle biopsies from a specific group of women with PCOS, which may not fully represent the diverse population of individuals with this condition. The in vitro experiments conducted on muscle cells may not capture the complexity of the in vivo environment, which can also be altered by factors such as hormones and other homeostatic processes in the body over time.
Zhao et al. (2017) [25]	175 women with PCOS and 127 healthy controls	*PPARGC1A* promoter hypermethylation (36.5% in PCOS vs. 26.3% in controls)	HOMA-IR: PCOS (2.54 ± 0.12) vs. controls (1.98 ± 0.06), P = 0.0054 (significant). Insulin: PCOS (11.64 ± 0.51 mU/L) vs. controls (9.26 ± 0.26 mU/L), P = 0.0032 (significant). Total testosterone: PCOS (1.22 ± 0.05 nmol/L) vs. controls (0.81 ± 0.05 nmol/L), P < 0.0001 (significant). Triglycerides: PCOS (1.68 ± 0.07 mmol/L) vs. controls (1.09 ± 0.05 mmol/L), P < 0.002 (significant). HDL-C: PCOS (1.32 ± 0.02 mmol/L) vs. controls (1.30 ± 0.02 mmol/L), P = 0.69 (not significant).	Women with PCOS exhibited significantly higher *PPARGC1A* promoter methylation and lower mtDNA content; *PPARGC1A* hypermethylation correlated with IR, waist circumference, and triglycerides. Lower mtDNA content was associated with increased metabolic risk. PCOS patients with metabolic syndrome had greater epigenetic alterations.	*PPARGC1A* promoter hypermethylation was linked to mitochondrial dysfunction and metabolic dysregulation. Higher methylation levels correlated with increased IR and triglyceride levels. Reduced mtDNA content was inversely associated with metabolic function in PCOS.	The cross-sectional design prevents the establishment of direct causal relationships between *PPARGC1A* promoter methylation, mtDNA content, and metabolic risk in women with PCOS. Although the study controlled for BMI, it did not account for other potential confounders such as diet, physical activity, or medication use. The population being investigated included women with PCOS from a specific university hospital, which may not be representative of all women with PCOS.
Jones et al. (2015) [26]	23 women with PCOS and 13 healthy controls	Hypomethylated: Luteinizing hormone/choriogonadotropin receptor (*LHCGR),* hypermethylated: *INSR*, *RAB5B*	Total testosterone: PCOS (32 ng/dL) vs. controls (28 ng/dL), P = 0.075 (not significant). Free testosterone: PCOS (4.9 pg/dL) vs. controls (2.3 pg/dL), P = 0.002 (significant); FBG: PCOS (88.5 mg/dL) vs. controls (74.5 mg/dL), P = 0.009 (significant); FINS: PCOS (9 μIU/mL) vs. controls (9.5 μIU/mL), P = 0.50 (not significant); HOMA2-IR: PCOS (0.99) vs. controls (1.09), P = 0.88 (not significant).	*LHCGR* was overexpressed in non-obese PCOS women, correlating with reduced methylation; I*NSR* was underexpressed in obese PCOS women, correlating with increased methylation; *RAB5B* was underexpressed in PCOS and had increased methylation at specific CpG sites. Findings suggest different genetic and epigenetic mechanisms driving PCOS in obese and non-obese women.	Hypomethylation of *LHCGR* was linked to increased expression, driving excess androgen secretion in non-obese PCOS patients. Hypermethylation of *INSR* correlated with reduced expression, contributing to IR in obese PCOS patients. Methylation changes in *RAB5B* suggest a role in altered insulin signaling and inflammation in PCOS.	A relatively small sample size, particularly in the methylation analysis. The study focused on subcutaneous adipose tissue, which may not fully represent the broader pathophysiology of PCOS; DNA methylation and expression patterns may vary depending on the tissues being examined. The study's cross-sectional design does not allow for direct causal relationships.
Gao et al. (2024) [27]	PCOS with IR = 13, PCOS with HA = 12, PCOS without IR or HA = 6, controls = 28 (total = 59)	TGF-β1 promoter hypomethylation at CpG4 and CpG7	HOMA-IR: PCOS-IR (4.51 ± 2.37) vs. controls (2.27 ± 0.86), P = 0.002 (significant); FINS: PCOS-IR (19.5 ± 8.77 µU/mL) vs. controls (9.56 ± 2.9 µU/mL), P < 0.001 (significant). Total testosterone: PCOS-HA (2.1 ± 0.69 nmol/L) vs. controls (1.06 ± 0.42 nmol/L), P < 0.001 (significant); BMI: PCOS-IR (23.92 ± 3.74) vs. controls (20.68 ± 2.40), P = 0.002 (significant).	TGF-β1 promoter hypomethylation at CpG4 and CpG7 was associated with IR in PCOS patients. Lower methylation levels correlated with higher HOMA-IR and FINS. No significant correlation between TGF-β1 methylation and mRNA/protein expression. Higher testosterone levels were observed in the high-methylation PCOS group.	Hypomethylation of TGF-β1 CpG4 and CpG7 was linked to increased IR. Methylation rate was negatively correlated with FINS and HOMA-IR. Methylation rates increased with age, potentially affecting PCOS risk.	The study focused on three generations of family members with PCOS and IR, limiting its generalizability to other populations or individuals with different phenotypes of PCOS. The study was not longitudinal and did not depict methylation changes over time, which might correlate with PCOS progression or the effectiveness of potential interventions. In comparison to prior literature, some studies demonstrate a clear correlation between TGF-β1 expression and markers like testosterone levels or IR, while others find weak or no associations.
Shen et al. (2013) [28]	10 PCOS patients: 5 with IR, 5 non-IR (NIR), and 5 healthy controls	79 differentially methylated genes between IR and NIR PCOS patients; 40 differentially methylated genes in PCOS vs. controls	HOMA-IR: PCOS-IR (3.18 ± 0.67) vs. PCOS-NIR (1.87 ± 0.55) vs. controls (1.53 ± 0.22), P < 0.05 (significant); FINS: PCOS-IR (13.4 ± 3.51 mIU/mL) vs. PCOS-NIR (8.42 ± 3.08 mIU/mL) vs. controls (6.54 ± 0.79 mIU/mL), P < 0.05 (significant). Total testosterone: PCOS-IR (3.25 ± 0.51 nmol/L) vs. PCOS-NIR (3.59 ± 0.58 nmol/L) vs. controls (1.67 ± 0.52 nmol/L), P < 0.05 (significant); SHBG: PCOS-IR (18.7 ± 7.99 nmol/L) vs. PCOS-NIR (30.3 ± 6.02 nmol/L) vs. controls (63.1 ± 19.8 nmol/L), P < 0.05 (significant); BMI: PCOS-IR (26.6 ± 0.77) vs. PCOS-NIR (21.3 ± 1.11) vs. controls (19.7 ± 2.41), P < 0.05 (significant).	PCOS-IR and PCOS-NIR exhibit distinct DNA methylation profiles, suggesting different epigenetic mechanisms; 79 genes were differentially methylated between PCOS-IR and PCOS-NIR patients, and 40 genes were differentially methylated in PCOS vs. controls; *CEBPB* methylation was significantly different between PCOS-IR and PCOS-NIR, indicating a role in IR. Differentially methylated genes were linked to immune response, inflammation, and metabolic pathways.	*CEBPB* hypomethylation in PCOS-IR was associated with increased IR. Differential methylation of genes involved in immune response and cytokine signaling suggests epigenetic regulation of inflammation in PCOS. Cancer-related pathways were differentially methylated in PCOS vs. controls, indicating potential long-term risks.	The sample size is relatively small, with only 10 PCOS patients and 5 controls, which may limit the generalizability of the findings. The lack of diversity in the sample population may also affect the applicability of the results to broader populations. The study did not account for potential confounding factors such as diet, lifestyle, or environmental exposures, which could influence methylation profiles. The reliance on peripheral blood samples might not fully capture tissue-specific methylation patterns.
Furtado et al. (2024) [29]	56 PCOS patients (30 resistance training, 26 aerobic training)	Genome-wide DNA methylation increased after both resistance and aerobic exercise. Resistance training altered methylation in all CpG island contexts (islands, shores, shelves, and open sea), while aerobic training altered CpG shores and open sea; 6 DMRs were noted in resistance training, and 14 were found in aerobic training; all hypermethylated.	BMI: No significant changes after either training type. Waist circumference: Reduced in both resistance and aerobic training (P = 0.022 and P = 0.014, respectively). Testosterone: Lower after resistance (93.17 ± 38.62 to 76.00 ± 27.23 ng/dL, P = 0.012) and aerobic training (109.19 ± 42.62 to 87.19 ± 32.8 ng/dL, P = 0.003). HDL: Decreased only after resistance training (P = 0.032). Triglycerides, FBG, FINS, and HOMA-IR showed no significant changes.	Resistance and aerobic training increased global DNA methylation, suggesting a role in PCOS management. Resistance training altered methylation in all genomic regions, while aerobic training primarily affected CpG shores and open sea. Epigenetic changes may contribute to improved metabolic and hormonal outcomes in PCOS.	Resistance training led to hypermethylation in genes associated with metabolic and hormonal regulation (e.g., *WRNIP1, NR2C2AP, DSCR9). *Aerobic training resulted in hypermethylation in genes linked to ribosomal function and RNA processing (e.g., *RPS27, RPL35A, RIOK2*). Epigenetic modifications from both exercise types may enhance genomic stability and metabolic function in PCOS.	The small sample size could limit the generalizability of the findings. Additionally, the absence of a control group without exercise limits the ability to definitively attribute the observed changes in DNA methylation and metabolic outcomes solely to the exercise interventions.

Discussion

**Figure 2:**
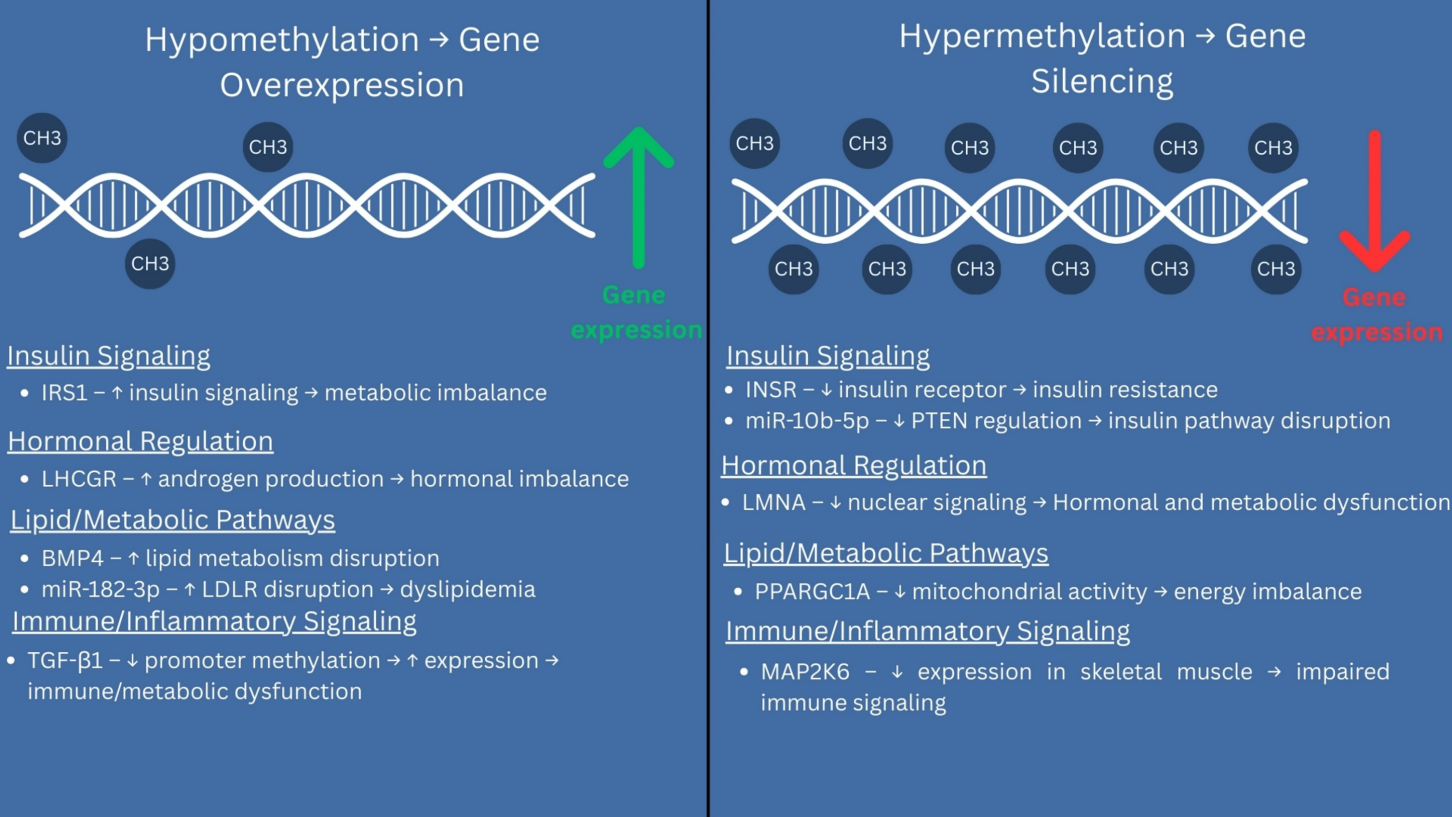
Hypo- and hypermethylation patterns in PCOS and their pathophysiological effects Image Credit: Lily Tehrani. This figure illustrates the role of DNA methylation in the pathophysiology of PCOS. Hypermethylation of genes such as *INSR, LMNA, PPARGC1A*, and miR-10b-5p leads to reduced gene expression, contributing to IR, mitochondrial dysfunction, and hormonal imbalance. Conversely, hypomethylation of *IRS1, BMP4, LHCGR,* TGF-β1, and miR-182-3p results in overexpression, promoting excess androgen production, disrupted lipid metabolism, and immune pathway dysregulation. These gene-specific methylation changes influence four major biological pathways: insulin signaling, hormonal regulation, lipid metabolism, and immune/inflammatory response, all of which contribute to the heterogeneous clinical presentation of PCOS. *IRS1*: Insulin Receptor Substrate 1; *LMNA*: lamin A/C; *PPARGC1A*: peroxisome proliferator-activated receptor gamma coactivator 1-alpha; miR-10b-5p: microRNA-10b-5p; *BMP4*: bone morphogenetic protein 4; *LHCGR*: luteinizing hormone/choriogonadotropin receptor; TGF-β1: transforming growth factor beta 1; miR-182-3p: microRNA-182-3p.

PCOS is a multifactorial endocrine disorder influenced by genetic, epigenetic, metabolic, and environmental factors. Among its most prominent characteristics are metabolic dysregulation and IR, which have been found to play a central role in its pathogenesis. Recent studies have investigated the role of epigenetic modifications, particularly DNA methylation, in mediating gene expression changes associated with insulin signaling, glucose metabolism, and hormonal balance in PCOS. The evidence supports that the altered methylation of specific genes is involved in the etiology and pathogenesis of PCOS, serving as potential biomarkers. Figure 2 summarizes key genes affected by hypo- and hypermethylation in PCOS and their downstream effects on IR, hormonal imbalance, lipid metabolism, and immune signaling.

Hypermethylation of the *INSR* gene was a common finding across multiple studies. *INSR*, which encodes the insulin receptor, has a crucial role in insulin signaling, which promotes glucose uptake [30]. In two studies, the hypermethylation of *INSR* was found to be associated with reduced expression of *INSR* and increased IR in obese PCOS patients [20,26]. These findings suggest that altered DNA methylation patterns and reduced *INSR* function contribute to the development of IR and metabolic dysfunction. Similarly, hypermethylation of the *LMNA* gene in PCOS patients was found to contribute to elevated FINS, glucose, and testosterone levels [19]. *LMNA* contributes to nuclear structural integrity and chromatin organization, with dysregulation in its expression suggesting a possible link between nuclear dysfunction and metabolic imbalance [31]. 

Another key gene, *PPARGC1A*, is an essential regulator of mitochondrial biogenesis and metabolic function [32]. *PPARGC1A* was found to be hypermethylated in the leukocytes of PCOS patients. This epigenetic alteration was associated with reduced mtDNA content, which triggered higher insulin levels, increased waist circumference, and hypertriglyceridemia [25]. These findings demonstrate the importance of understanding DNA methylation changes regarding the development of insulin sensitivity and energy metabolism in PCOS. 

On the other hand, alterations that reduce DNA methylation have also been found to affect IR and metabolic dysregulation. Hypomethylation of the *IRS1* and *BMP4* genes was observed in GCs of PCOS patients [22]. Given the link between mitochondrial dysfunction and metabolic abnormalities, this further suggests the notion that epigenetic alterations can lead to metabolic dysregulation. Hypomethylation led to increased expression of *IRS1* and *BMP4*, ultimately disrupting insulin-signaling pathways and altering lipid metabolism, respectively. 

Moreover, the methylation of TGF-β1, which is primarily involved in fibrosis, inflammation, and hormonal regulation, was also found to be altered in PCOS patients [33]. In particular, hypomethylation at the CpG4 and CpG7 sites within the TGF-β1 promoter was linked to IR as patients had higher HOMA-IR scores and FINS levels [27]. Although its exact role in PCOS is still under investigation, TGF-β1 methylation changes appear to have an influential role in the pathways connected to metabolic dysregulation. 

The regulation of non-coding RNAs, such as miRNAs, has also been found to be impacted by methylation patterns. Hypermethylation and downregulation of miR-10b-5p, miR-23a-3p, and miR-127-3p lead to increased expression of target genes such as *PTEN* [21]. *PTEN*, a negative regulator of the PI3K/Akt pathway, plays an important role in insulin signaling and metabolic regulation. Hypermethylation and downregulation of miR-10b-5p, miR-23a-3p, and miR-127-3p lead to increased expression of target genes such as PTEN [23]. PTEN, a negative regulator of the PI3K/Akt pathway, suppresses insulin signaling when overexpressed, contributing to IR. Conversely, hypomethylation of miR-182-3p, miR-140-5p, and miR-200b-5b led to disruptions of ovarian function and alterations in lipid metabolism with negative effects on LDLR, OLR1, and other key metabolic regulators. Several layers of regulation are affected as DNA methylation alters miRNA expression, which in turn regulates genes involved in insulin signaling [23]. 

Furthermore, studies on the *LHCGR* and *RAB5B* genes revealed obesity-dependent methylation changes that influenced gene expression. Researchers found that excess androgen production was linked to *LHGCGR* hypomethylation and overexpression in non-obese PCOS patients [26]. *RAB5B* and *INSR*, however, demonstrated hypermethylation and downregulation, highlighting their role in altered insulin signaling and inflammation. These findings suggest the complex nature of PCOS pathogenesis and how obesity can influence the way various genes are expressed. 

In addition, a similar study analyzing the skeletal muscle biopsies from women with PCOS identified several differentially methylated genes involved in IR, including krüppel-like factor 10 (*KLF10*), *COL1A1*, *MAP2K6*, titin (*TTN*), and *NBPF20.* Insulin was found to increase *KLF10* expression, while testosterone was found to downregulate the *COL1A1* and *MAP2K6* expression. Moreover, both C-peptide levels and the C-peptide indices were significantly higher in women with PCOS as compared to controls. This relationship between C-peptide and PCOS further supports the fact that metabolic dysregulation and IR were positively correlated with the expression of genes *KLF10* and *TTN, *and negatively correlated with genes *COL1A1* and *NBPF20*. These findings suggest a connection between muscle metabolism and PCOS, as altered gene expression was associated with increased C-peptide levels and altered adipocyte size [24]. 

Lifestyle factors, such as resistance and aerobic training, have also led to changes in DNA methylation patterns in PCOS patients [29]. Resistance training was linked to hypermethylation of genes, such as *WRNIP1* and *NR2C2AP*, which are both involved in metabolic regulation, while aerobic training was found to influence expression of genes associated with RNA processing and ribosomal function. The improved testosterone levels and waist circumference of patients suggest that non-pharmacological options can influence epigenetic modulation. 

The findings from these studies, specifically the disruption of insulin signaling observed in *IRS1* and *INSR*, are largely consistent with prior research on the epigenetic modifications in PCOS with IR. While *PPARGC1A's* role in mitochondrial dysfunction and metabolic risk is currently emerging, further exploration of the role of DNA methylation of these genes needs to be further investigated in relation to metabolic dysregulation. DNA methylation can serve as a valuable biomarker for diagnostics and for understanding the complex disease mechanisms of PCOS. Further investigations on genome-wide DNA methylation patterns are necessary for understanding how these epigenetic alterations can contribute to PCOS disease progression. An emphasis on longitudinal studies can also help clarify the trajectory of PCOS by examining how these methylation patterns evolve. 

Despite the valuable insights provided, the studies reviewed had several limitations, with small sample sizes being the most common. Additionally, DNA methylation patterns would vary across different tissue types at different times. Therefore, creating more studies that analyze similar tissue types under specific time markers would be beneficial. Moreover, most studies were cross-sectional or case-control in their design, making it difficult to determine whether changes in DNA methylation patterns were caused by IR or are a result of it. This limits the causality of DNA methylation changes in relation to IR and metabolic dysregulation in PCOS patients. Larger-scale studies are essential to confirm and better understand these limitations. Additionally, several studies did not fully validate their findings. For example, some studies conducted a bioinformatic analysis without confirming their results in vitro or in patient samples [22], while others only partially validated their results through statistical or internal comparison [25-29]. This limitation reduces the strength of their results and highlights the need for future research that includes functional validation, such as transcriptome or protein-level analysis. Furthermore, studies that explore the effects of lifestyle interventions, such as exercise and diet, on DNA methylation patterns could offer adjuvant therapies to improve insulin sensitivity and metabolic health in PCOS patients. 

Overall, these findings have strongly supported the idea that DNA methylation serves a critical role in the pathogenesis of IR and metabolic dysfunction in PCOS. Further studies should aim to analyze these epigenetic changes in larger PCOS populations and utilize this as potential insight for therapeutic targets with gene modulation therapy, lifestyle modulation, or a combination of both. 

## Conclusions

Overall, the current body of literature has shown that DNA methylation alterations are interconnected with IR through dysregulation of insulin signaling pathway genes (*INSR, IRS1*), lipid metabolism genes (*PPARGC1A*), mitochondrial function, and hormonal pathways (*AMHRII, *TGF-β1). These epigenetic alterations are associated with clinical features of PCOS such as HA and metabolic derangement. However, causal interpretations are constrained by observational study designs. The findings suggest that hypomethylation of certain genomic regions can upregulate genes for IR and adipose dysfunction, while hypermethylation of promoters (e.g., *PPARGC1A*) suppresses mitochondrial and metabolic function. In addition, miRNAs and inflammatory pathways also crosstalk with methylation changes to exacerbate PCOS phenotypes. Therapeutic potential lies in targeting reversible epigenetic alterations, such as exercise and insulin sensitizers, like metformin, to normalize metabolic gene methylation patterns. Additionally, modulation of *PPARGC1A* or *IRS1 *may directly improve insulin sensitivity. Future research priorities should include longitudinal studies to establish causality and the exploration of chromatin-modifying therapies for PCOS-specific epigenetic markers.
